# The Effects of a Co-Application of Menthol and Capsaicin on Nociceptive Behaviors of the Rat on the Operant Orofacial Pain Assessment Device

**DOI:** 10.1371/journal.pone.0089137

**Published:** 2014-02-18

**Authors:** Ethan M. Anderson, Alan C. Jenkins, Robert M. Caudle, John K. Neubert

**Affiliations:** 1 Department of Oral and Maxillofacial Surgery, University of Florida College of Dentistry, Gainesville, Florida, United States of America; 2 Department of Neuroscience, University of Florida College of Medicine, McKnight Brain Institute, Gainesville, Florida, United States of America; 3 Department of Orthodontics, University of Florida, Gainesville, Florida, United States of America; Tokai University, Japan

## Abstract

**Background:**

Transient receptor potential (TRP) cation channels are involved in the perception of hot and cold pain and are targets for pain relief in humans. We hypothesized that agonists of TRPV1 and TRPM8/TRPA1, capsaicin and menthol, would alter nociceptive behaviors in the rat, but their opposite effects on temperature detection would attenuate one another if combined.

**Methods:**

Rats were tested on the Orofacial Pain Assessment Device (OPAD, Stoelting Co.) at three temperatures within a 17 min behavioral session (33°C, 21°C, 45°C).

**Results:**

The lick/face ratio (L/F: reward licking events divided by the number of stimulus contacts. Each time there is a licking event a contact is being made.) is a measure of nociception on the OPAD and this was equally reduced at 45°C and 21°C suggesting they are both nociceptive and/or aversive to rats. However, rats consumed (licks) equal amounts at 33°C and 21°C but less at 45°C suggesting that heat is more nociceptive than cold at these temperatures in the orofacial pain model. When menthol and capsaicin were applied alone they both induced nociceptive behaviors like lower L/F ratios and licks. When applied together though, the licks at 21°C were equal to those at 33°C and both were significantly higher than at 45°C.

**Conclusions:**

This suggests that the cool temperature is less nociceptive when TRPM8/TRPA1 and TRPV1 are co-activated. These results suggest that co-activation of TRP channels can reduce certain nociceptive behaviors. These data demonstrate that the motivational aspects of nociception can be influenced selectively by TRP channel modulation and that certain aspects of pain can be dissociated and therefore targeted selectively in the clinic.

## Introduction

The transient receptor potential (TRP) family of cation channels are involved in the perception of hot and cold temperatures and pain [1], [2]. The transient receptor potential vanilloid receptor 1 (TRPV1) is activated by heat (>43°C), low pH, and capsaicin [3] while the transient receptor potential melastatin 8 receptor (TRPM8) is responsible for detecting cool temperatures (<23°C) and can be activated by menthol [4], [5]. The transient receptor potential subfamily A1 receptor (TRPA1) can also be activated by menthol and is responsible for the detection of cooler temperatures [6]. Expression changes in these proteins occur in humans with pain syndromes like diabetic neuropathy [7] as well as animal models of neuropathic pain like spared nerve injury (SNI) [8] and chronic constriction injury (CCI) [9], [10] suggesting that studying their modulation could be beneficial to treating chronic pain in the future.

TRPV1 and TRPM8/TRPA1 can modulate each other's effects on heat and cold pain. Menthol enhances cold pain and reduces heat pain [11]. It also has a “cooling” effect on warm temperatures [12]. Capsaicin evokes “burning” sensations in humans which can alter cold perception [13]. When applied alone, capsaicin reduces heat pain thresholds but a combination of menthol and capsaicin increases cool detection thresholds [13], [14]. Also, menthol increases cold pain thresholds alone, but this is attenuated when capsaicin is added [13]. So it appears that some interaction is occurring between these two types of receptors and/or the neurons they are on.”

In order to investigate the effects of TRPV1 and TRPM8/TRPA1 co-activation on the behavior of awake, functioning rats we used an orofacial operant assay which is sensitive to heat, cold, and mechanical nociception [15]–[17]. We previously demonstrated that orofacial pain assays can detect the modulation of thermal nociception with TRP channel agonists like capsaicin, resiniferatoxin, menthol, and icillin [17]–[20]. We hypothesized that menthol would reduce heat-induced nociceptive behaviors and capsaicin would reduce cold nociceptive behaviors.

## Methods

All facilities are accredited by the Association for Assessment and Accreditation of Laboratory Animal Care and all procedures were approved by the University of Florida's Institutional Animal Care and Use Committee.

Sixteen male, hairless Sprague-Dawley rats (Charles River, Raleigh, NC) weighing 400–600 g were used for all procedures. Rats were housed in pairs in 22°C temperature and 31% humidity controlled rooms with a 12-hour light/dark cycle (Lights were on during the hours of 6am-6pm and behavioral sessions were performed between 8am and 12pm.). Rats had free access to food and water except when fasted for behavioral testing.

**Figure 1:**
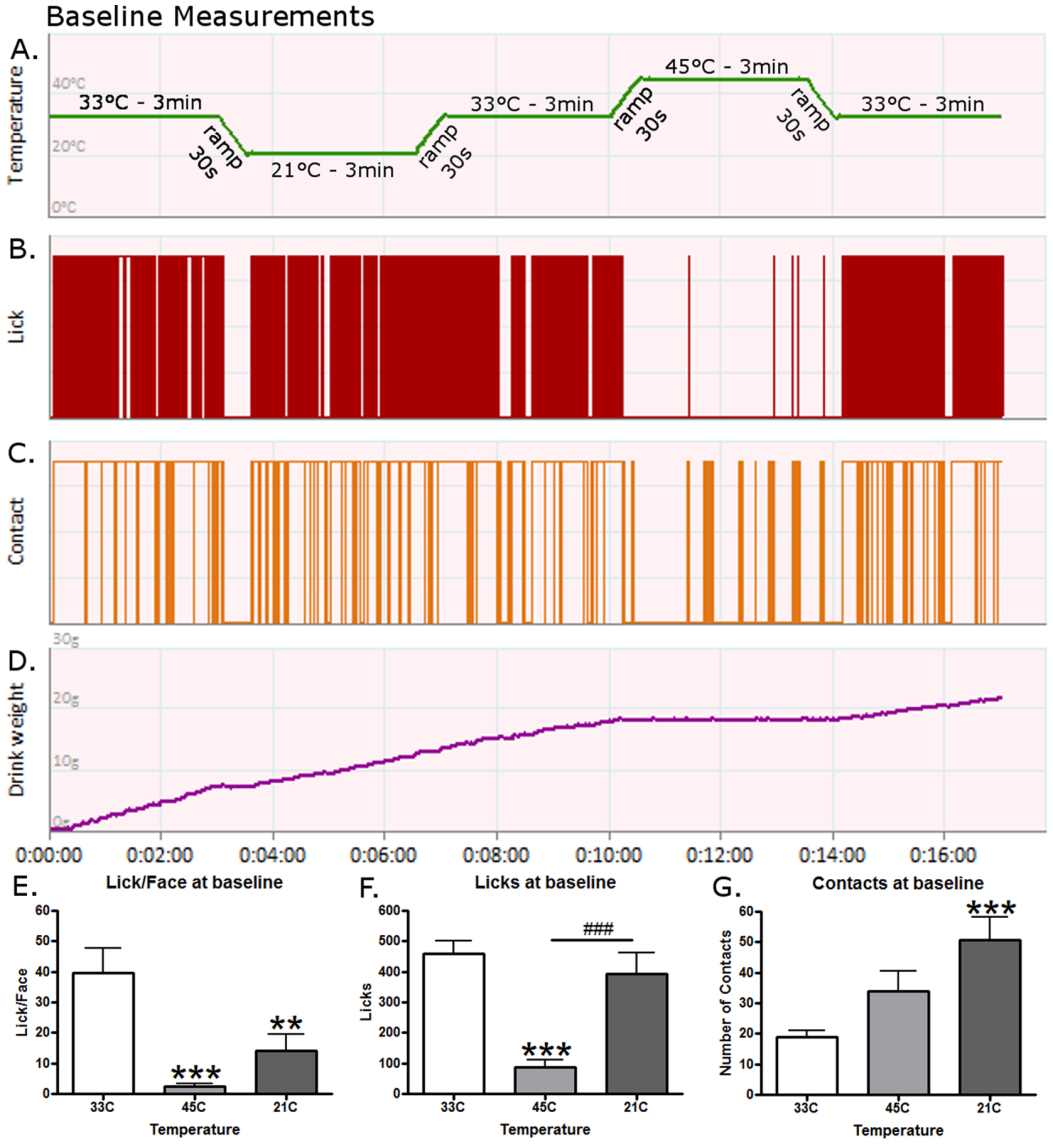
The effects of hot and cool temperatures on operant nociceptive behavior. A single, representative rat's responses to different stimuli under baseline conditions during a (A) temperature ramping protocol for (B) licking behavior, (C) contact behavior, and (D) reward intake. (E) The Lick/Face ratio was dependent on temperature. 45°C and 21°C temperatures caused lower Lick/Face ratios (post-hoc tests, p<0.001 and p<0.01). (F) Licking events were altered by temperature. Rats licked less at 45°C compared to 33°C and 21°C (p<0.001 and p<0.001). (G) Contact behavior was altered by temperature. More contacts were made at 21°C than at 33°C (p<0.001). An ** and *** represent p-values less than 0.01 and 0.001 respectively for a Bonferroni's post-hoc test when comparing behavior to 33°C after a One-Way ANOVA. A ### represents a p-value less than 0.001 when comparing 21°C to 45°C with Bonferroni's. N = 16 for graphs E-G.

The methods of Anderson et al. [21] were used for this study. Briefly, rats were trained on the Orofacial Pain Assessment Device (OPAD, Stoelting, Co., Wood Dale, IL) to press their faces into peltier devices whose temperature can be heated or cooled to aversive temperatures. If they performed this task they received access to a reward bottle filled with diluted sweetened condensed milk (1∶2, milk:water). Food-fasted rats (15+/−1 hrs) were trained at a neutral temperature (33°C) until they would make at least 2,000 licks during a 20 min session as a standard. If an animal did not meet this criterion, it was excluded from the study. The selection of the temperatures used was based on previous work our group has done in this area. Rodents will have the largest L/F ratios and the longest bouts of reward consumption at temperatures between 30–37°C [10], [15]–[28] suggesting that these are the least nociceptive/aversive temperatures. Rats appear to like temperatures close to 33°C as they provide warm, but not hot sensations. Next, baseline measurements were obtained for three temperatures within a single session. This was accomplished by ramping the temperature several times over the course of a 17 min session as illustrated in **Figure 1A**.

After baseline data was collected each rat received all of the agonist cream treatments described below and thus served as its own control. The same 17 min ramping protocol was repeated but with the addition of either 1.25% menthol (half velvachol (Healthpoint Biotherapeutics, Fort Worth, TX), half 2.5% menthol cream (IcyHot Vanishing Scent, Chattem, Inc., Chattanooga, TN)), 0.05% capsaicin (half velvachol, half 0.1% capsaicin cream (Capzacin HP, Chattem, Inc., Chattanooga, TN)), or both (half 2.5% menthol cream, half 0.1% capsaicin cream). Rats were first anesthetized with 1–2.5% isoflourane gas and the cream mixture was placed on both cheeks. After five minutes it was washed off with water and then wiped with an alcohol pad. Rats were tested 30 min later. These concentrations of menthol and capsaicin were chosen as they are commercially available to the general public for over-the-counter pain relief. Thus these results can be more easily reproduced by others in the field as the preparation of the application cream should remain constant from lab to lab.

Data were analyzed within session with repeated measures One-Way ANOVA followed by Bonferroni's post-hoc test. The three 33°C time periods were averaged into one 3 min session and this was compared to the 21°C and 45°C time period (both 3 min). Behavior during the ramping times was not analyzed. If a rat did not perform during a session a zero value was used for the Lick/Face value. Paired t-tests were also performed in some cases as noted below. For all analyses, a p-value of less than 0.05 was considered significant. GraphPad Prism 4 software was used for all analyses (La Jolla, CA).

Two additional experiments were performed to further examine some of the results described below. The first was on the effects of menthol at a colder temperature of 7°C. The same protocols were used as stated above except that the schedule now dropped to 7°C for 3 min instead of 21°C, this required the ramping times to be increased from 30 s to 1 min and therefore the total session time was increased from 17 min to 18 min. A baseline session and a menthol treated session were both performed and the data are presented below. Grubbs' test was used to remove any outliers before paired t-tests were performed to assess significance. The second experiment involved placing the 45°C segment earlier in the protocol. For this protocol, the 3 min 33°C session occurred, followed by a 30 s ramp, then the 45°C ensued. Rats were run as described above for a baseline session and a capsaicin treated session. Grubbs' test and paired t-tests were again used to determine significant effects.

## Results

Representative behavior for a single animal during baseline readings is displayed in **Figure 1B–D**. Reward licking events (Licks) are denoted as red bars as illustrated in **Figure 1B**. For this animal, licking events were frequent at all times other than 45°C. **Figure 1C** illustrates the stimulus contact behavior. Note that each time there is a licking event a contact is being made as displayed. However, an animal can contact the stimulus but not lick, indicating an unsuccessful attempt which is characteristic of a nociceptive response [15]. For example, the increased contacts observed in the 45°C session demonstrate that the rats are making attempts for the reward. However, the heat is preventing them from staying on long enough to lick and this decreases their Lick/Face values. **Figure 1D** displays the reward consumption during the testing session. The data for all rats are quantified below. During baseline conditions the Lick/Face ratio was dependent on temperature (**Figure 1E**, F(2,15) = 11.44, p = 0.0002) as they were high at 33°C, but low at 45°C and 21°C comparatively (p<0.001 and p<0.01) indicating that 21°C and 45°C are nociceptive and/or aversive conditions to rats. The total number of licks were different however, as feeding behavior was altered by temperature (**Figure 1F**, F(2,15) = 17.30, p<0.0001), only the 45°C temperature was lowered compared to 33°C and 21°C (p<0.001 and p<0.001). This suggests that the 45°C temperature was more nociceptive and/or aversive than the 21°C as rats will barely attempt to obtain the reward at the hot temperature. At the cool temperature, they do alter their Lick/Face values, but still maintain high levels of reward consumption. Finally, contact behavior was also altered by temperature (**Figure 1G**, F(2,15) = 11.40, p<0.002) as the number at 21°C was higher than at 33°C (p<0.001). This demonstrates the change in behavior that rats underwent at 21°C. Because they found the stimulus aversive, they were forced to increase the number of times they went for the reward as a compensation mechanism.

**Figure 2:**
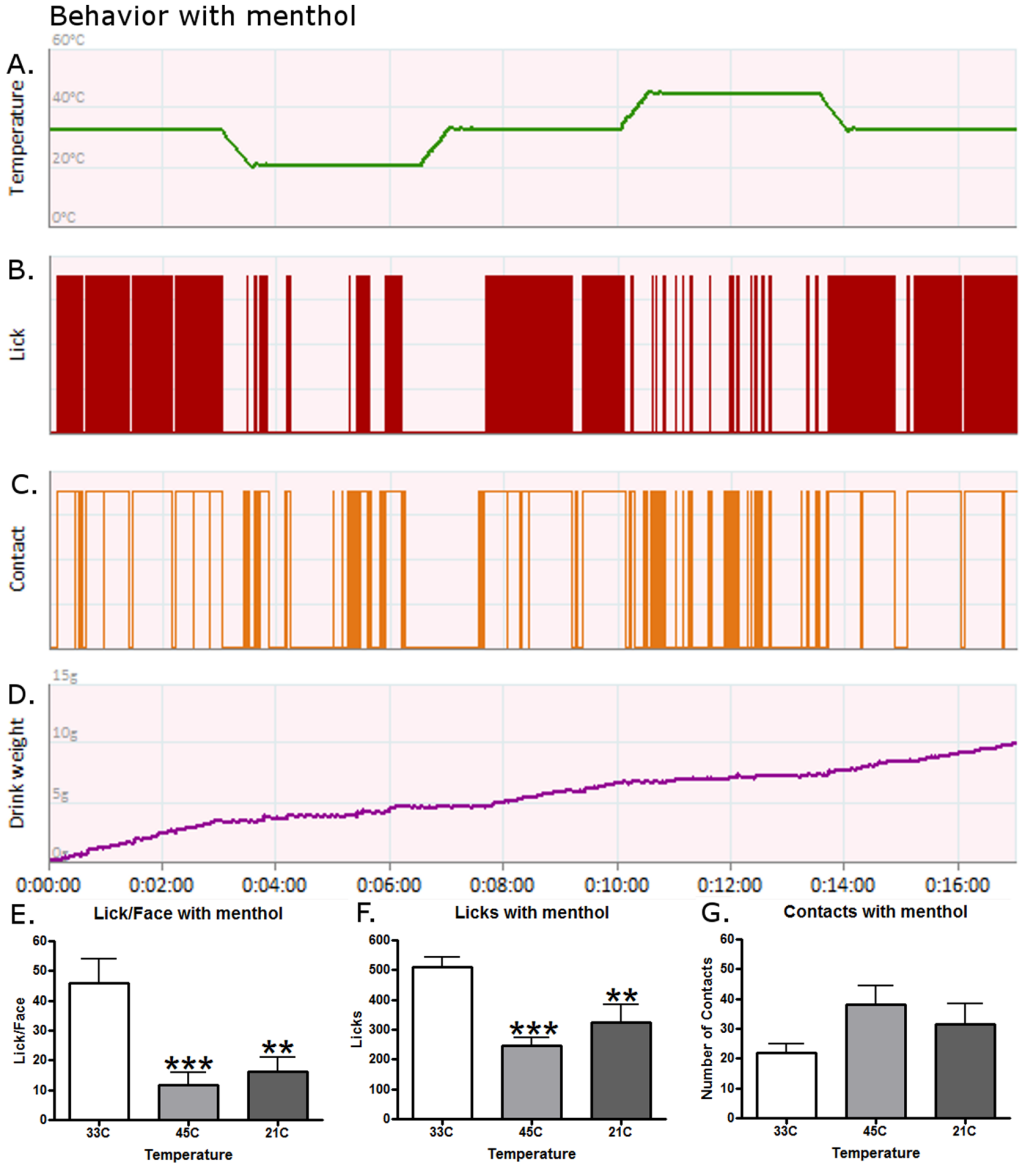
The effects of menthol on operant behavior. A single, representative rat's response after a menthol application during a (A) temperature ramping protocol for (B) licking behavior, (C) contact behavior, and (D) reward intake. (E) Lick/Face values were still altered by temperature. 45°C and 21°C still lowered this value (post-hoc tests, p<0.001 and p<0.01). (F) Licking behavior was altered by temperature differently than at baseline, both 45°C and 21°C were now lower than 33°C (p<0.001 and p<0.01). (G) No differences were observed for contacts with the menthol application. An ** and *** represent p-values less than 0.01 and 0.001 respectively for a Bonferroni's post-hoc test when comparing behavior to 33°C after a One-Way ANOVA. N = 16 for graphs E-G.

Representative behavior for a single rat after menthol application is displayed in **Figure 2B–C**. **Figure 2A** displays the ramping protocol again for ease of comparison and as displayed in **Figure 2B**, the licking decreased non-significantly at 21°C as compared to baseline, but significantly increased at 45°C as compared to baseline (paired t-test, t(15) = 4.800, p = 0.0002). **Figure 2C** demonstrates that fewer attempts to obtain the reward were made during the 21°C session and more were made at 45°C. **Figure 2D** illustrates the reward intake. Lick/Face values were not altered by menthol treatment. Similar to baseline measures, temperature still altered behavior (**Figure 2E**, F(2,15) = 12.93, p<0.0001) and 45°C and 21°C still lowered this value (p<0.001 and p<0.01). Licking behavior with menthol was different from baseline but temperature still had an effect (**Figure 2F**, F(2,15) = 16.72, p<0.0001). Although licking was still lower at 45°C compared to 33°C (p<0.001), 21°C was also lower (p<0.01). This was due to the mean number of licks at 45°C increasing with menthol and decreasing at 21°C. These two effects together had an effect such that there was no longer a significant difference between these two, unlike at baseline. Another effect of menthol was to reduce contacts at 21°C such that there were no longer any temperature differences for this aspect of the behavior (**Figure 2G**). Once again this was due to different effects at the two temperatures. The mean contact value was higher at 45°C and lower at 21°C so that there was no difference.

**Figure 3:**
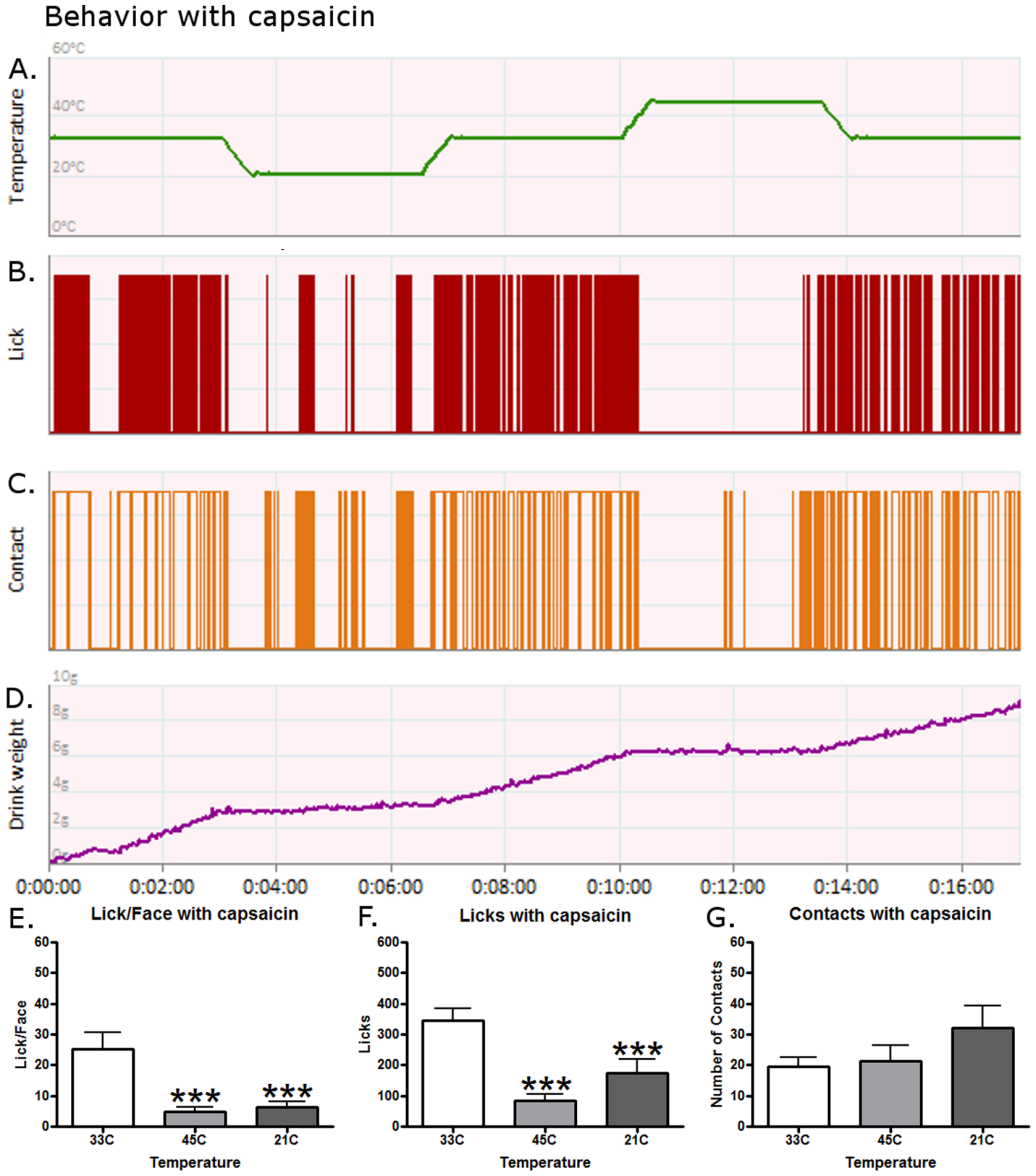
The effects of capsaicin on operant behavior. A single, representative rat's response to capsaicin during a (A) temperature ramping protocol for (B) licking behavior, (C) contact behavior, and (D) reward intake. (E) Temperature still had a significant effect on behavior with capsaicin application. The Lick/Face ratio was still lower at 45°C and 21°C than at 33°C (post-hoc tests, p<0.001 and p<0.001). (F) The number of licks was altered by temperature differently than at baseline. Licks at 33°C were higher than the other two temperatures (both p<0.001). (G) Contact behavior was no longer dependent on temperature when capsaicin was applied. An *** represents a p-value less than 0.001 for a Bonferroni's post-hoc test when comparing behavior to 33°C after a One-Way ANOVA. N = 16 for graphs E–G.

A single rat's behavior is displayed in **Figure 3B–D** after capsaicin application. Capsaicin altered behavior in a somewhat similar way to menthol during the ramping protocol (**Figure 3A**). Licks were significantly decreased for the 21°C period as compared to baseline (paired t-test, t(15) = 3.368, p = 0.0042) but not significantly altered at 45°C (**Figure 3B**) possibly due to a floor effect. Contacts were high for this rat as more attempts were needed at 33°C to obtain the reward (**Figure 3C**). The reward intake for this representative rat is displayed in **Figure 3D**. Capsaicin appears to have reduced the mean Lick/Face values at 33°C compared to baseline, but temperature still had a significant effect (**Figure 3E**) as the Lick/Face ratio was lower at 45°C and 21°C than at 33°C (p<0.001 and p<0.001). Capsaicin also reduced the number of licks (**Figure 3F**, F(2,15) = 25.89, p<0.0001) at 21°C substantially more than 33°C such that they were no longer significantly different from 45°C. Licks at 33°C were still higher than the other two temperatures (both p<0.001). Similar to the effects of menthol, contact behavior was no longer dependent on temperature (**Figure 3G**).

**Figure 4:**
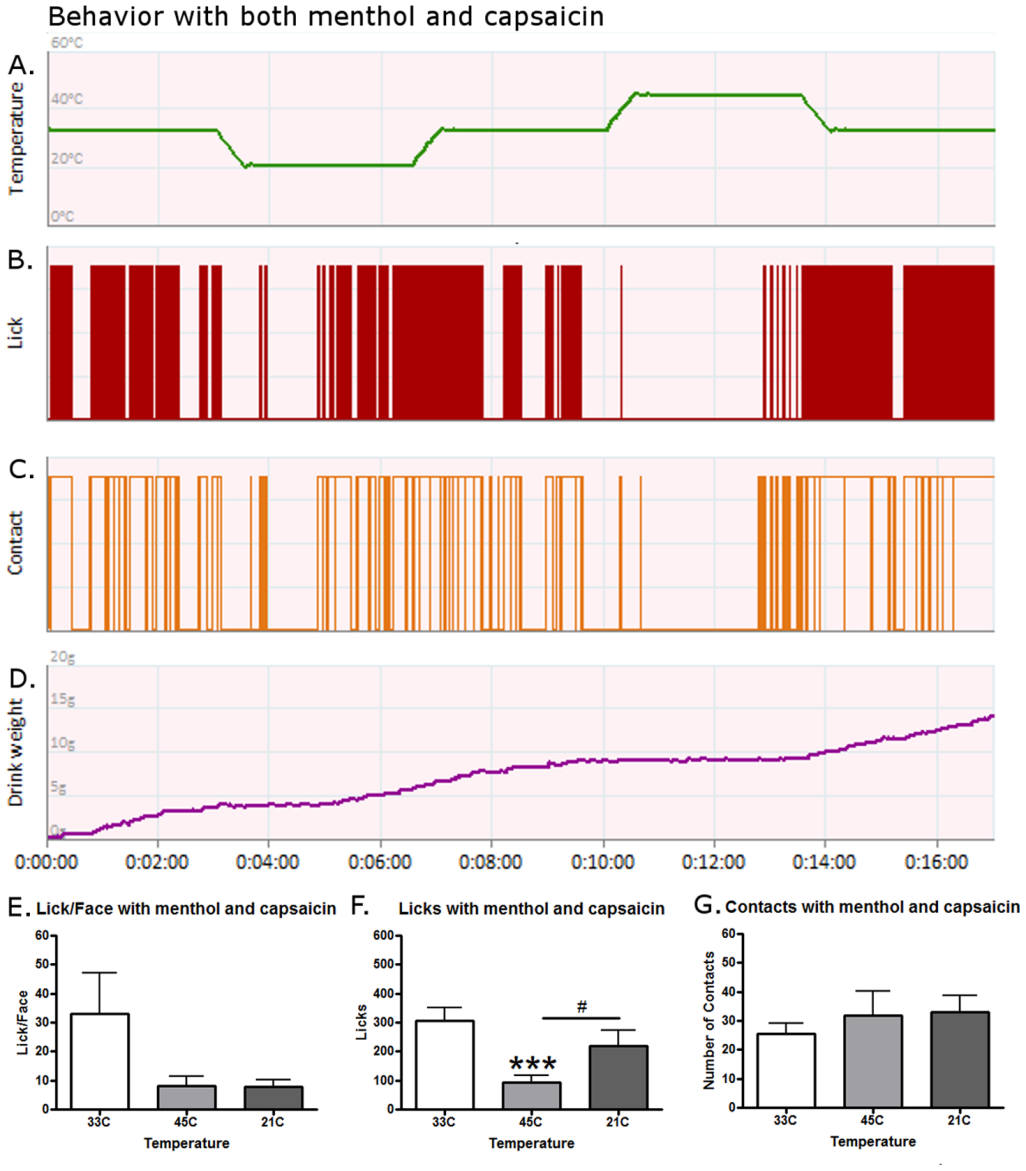
The effects of a co-application of menthol and capsaicin on operant behavior. A single, representative rat's response to menthol and capsaicin during a (A) temperature ramping protocol for (B) licking behavior, (C) contact behavior, and (D) reward intake. (E) Temperature still had a significant effect on the Lick/Face ratio when both capsaicin and menthol were applied although no post-hoc tests were significant. (F) Licking behavior was still temperature-dependent but the combination of menthol and capsaicin restored the original relationship for licking as now licking was equal between the 33°C and 21°C periods again and 45°C was again lower than both 33°C and 21°C (p<0.001 and p<0.05). (G) The number of contacts was again not dependent on temperature. An *** represent p-values less than 0.001 for a Bonferroni's post-hoc test when comparing behavior to 33°C after a One-Way ANOVA. A # represents a p-value less than 0.05 when comparing 21°C to 45°C with Bonferroni's. N = 16 for graphs E–G.

In our next experiment, we combined menthol and capsaicin to determine if the effects of one agonist would alter the other agonists' effects at the same ramping protocol (**Figure 4A**). Data for a single representative rat is displayed in **Figure 4B–D**. Licking was still low at 45°C, but relatively high at 21°C (**Figure 4B**). Contacts were similar between temperatures as many unsuccessful attempts were made during the 45°C session (**Figure 4C**). The reward intake for this representative rat is displayed in **Figure 4D**. Temperature still had a significant effect on the Lick/Face ratio when both capsaicin and menthol were applied (**Figure 4E**, F(2,15) = 4.129, p = 0.0261) although no post-hoc tests were significant. Licking behavior was still temperature-dependent (**Figure 4F**, F(2,15) = 11.86, p = 0.0002) and was similar to baseline measures as 45°C was lower than both 33°C (p<0.001) and 21°C (p<0.05). The number of contacts however, were again not dependent on temperature when the two drugs were co-applied (**Figure 4G**). These data suggest that an interaction has occurred when capsaicin and menthol are co-applied. While both agonists by themselves reduced licking events as compared to the 33°C temperature, when combined together rats licked just as much at 21°C as they did at 33°C suggesting a reduction of cold sensitivity, nociception, and/or aversion.

**Figure 5:**
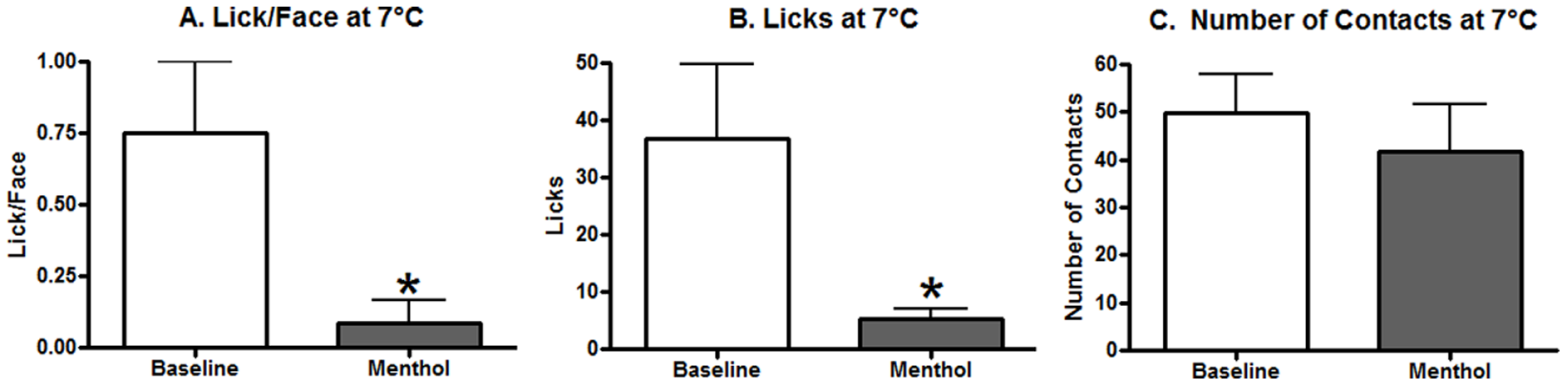
The effects of menthol on a cold 7°C temperature. (A) The Lick/Face ratio was significantly reduced by menthol application 30 mins before testing. (B) Licking behavior was also significantly reduced by menthol. (C) No effect was observed for contact behavior. A * represents a p-value of less than 0.05 with a paired t-test. N = 13 for all graphs.

We had hypothesized that menthol would decrease the licking behavior at the cool temperature of 21°C, and although it did decrease, this result was not statistically significant. We examined the effects of menthol on a colder temperature of 7°C to address this unexpected result. As illustrated in **Figure 5A**, the Lick/Face ratio was significantly decreased by menthol at 7°C (paired t-test, t(11) = 2.966, p = 0.0128). This effect was due to a significant reduction of licking behavior 7°C (**Figure 5B**, paired t-test, t(11) = 2.662, p = 0.0221) demonstrating the effects of menthol on altering consumption behavior and nociception at cold temperatures on this assay. No effect was observed for contact behavior (**Figure 5C**, paired t-test, t(11) = 0.7766, p = 0.4538).

**Figure 6:**
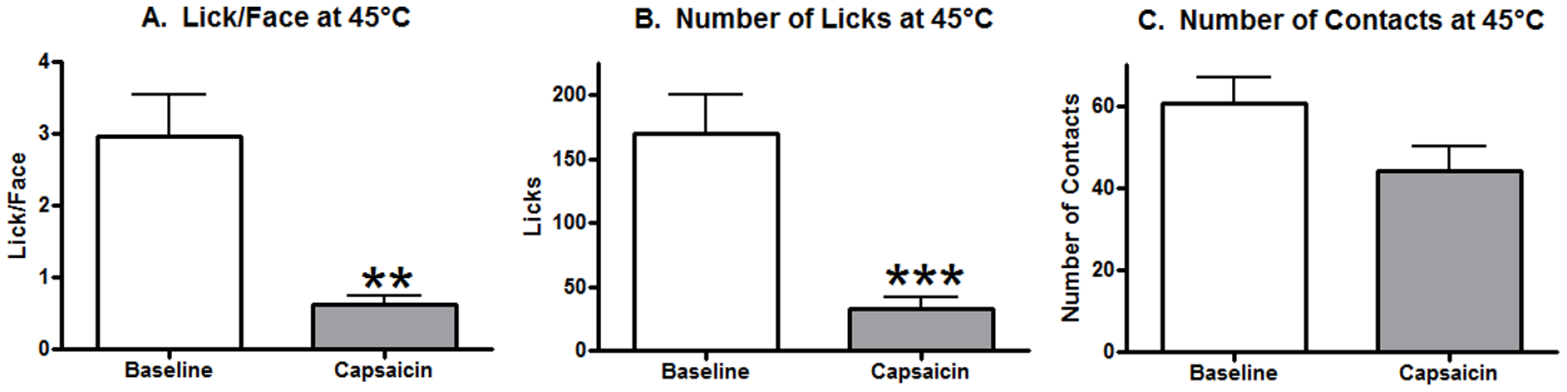
The effects of capsaicin on behavior at 45°C at 3.5 min instead of 10.5 min. (A) The Lick/Face ratio was significantly reduced by capsaicin application 30 mins before testing. (B) Licking behavior was also significantly reduced. (C) No effect was observed for contact behavior. A ** and *** represent p-values of less than 0.01 and 0.001 respectively, with a paired t-test. N = 14 for all graphs.

Since it is possible that satiety or sensitization to pain over the course of the 17 min protocol could have influenced the results at 45°C, we performed an additional experiment where the 45°C session occurred immediately after the first 33°C period. As illustrated in **Figure 6A**, the Lick/Face ratio was reduced by capsaicin at 45°C (paired t-test, t(13) = 3.999, p = 0.0015) when this temperature occurred earlier in the protocol. Licking behavior was also significantly reduced at 45°C (**Figure 6B**, paired t-test, t(13) = 4.343, p = 0.0008) but no significant effect was observed for contact behavior (**Figure 6C**, paired t-test, t(13) = 1.835, p = 0.0894).

## Conclusions

Nociceptive behaviors like the lick/face ratio, number of licks, and number of contacts were dependent on the temperature of the peltier devices on the OPAD at baseline. Consumption behavior (number of licks) was altered by the application of either menthol or capsaicin alone. When co-applied however, menthol and capsaicin reversed the effects on licks during a cool temperature of 21°C. This demonstrates that the co-activation of TRPM8/TRPA1 and TRPV1 channels produces a behavior that is different from activating either one alone. Different effects of temperature were observed for Lick/Face, licking behavior, and contact behavior at baseline on the OPAD. The non-neutral temperature conditions caused rats to alter their behavior from many licks per facial contact to few licks per facial contact at both 21°C and 45°C. This demonstrates that they perceive pain during these conditions. Nociception decreases the Lick/Face value on operant orofacial assays and analgesia can increase it [10], [15], [17]–[19], [24]. However, the 45°C condition had a greater impact on the rats' reward consumption behavior than 21°C. Licking was equally high between the 33°C and 21°C temperatures. So even though the rats' have a nociceptive and/or aversive response at 21°C, they continue to consume reward. A combination of increased number of contacts and a low Lick/Face ratio allows them to consume more reward at 21°C.

Some concerns have been raised about the use of 21°C as a nociceptive/aversive temperature. Although the 21°C temperature is similar to room temperature, in this situation a rat is pressing its hairless face into a metal bar at 21°C. This does produce a cool/cold effect similar to touching one's hand to a metal surface. This is likely more aversive to a rat as previous studies of ours have demonstrated that rats prefer warm temperatures to cold temperatures and that temperatures of 10°C and 24°C both reduce the Lick/Face ratio about 40% compared to baseline [17]. For this study we wanted to pick a “cooler” temperature rather than a “colder” temperature as we wanted to be able to detect a change with menthol application. Menthol altered the licking and contact behavior of the rats. The main effect was that there was no longer a difference between licks at 45°C and 21°C. These temperatures now had the same inhibiting effects on reward consumption. Licks at 21°C were now lower than at 33°C as well suggesting that menthol made this temperature more nociceptive and/or aversive. This effect was likely due to an increase in licking at 45°C and 33°C. The licks at these temperatures were higher than at baseline and this likely due to a cooling effect of menthol on hot/warm temperatures [11], [12]. Another possibility is that menthol could be producing these effects through direct inactivation of voltage-gated sodium channels or voltage-gated calcium channels. Menthol can block both of these types of channels which could lead to decreases in the excitability of heat sensing neurons. Behaviorally, this could result in antinociceptive/analgesic effects and explain the increases in licking behavior at 45°C and 33°C [29]–[33]. We had expected a decrease at 21°C due to menthol causing the cool temperature to become more aversive or nociceptive, perhaps because of menthol's ability to lower the cold threshold of TRPM8 [5]. However, the decrease observed was not statistically significant. This lack of a statistically significant effect of menthol at 21°C was unexpected. Since modest effects on behavior were observed, we hypothesize that future experiments with higher concentrations of menthol (ex. 10%) or colder temperatures (ex. 7°C) could produce an observable effect as we have previously reported on our operant orofacial assay [17].

We decided to pursue this hypothesis by using the same concentration of menthol but at a colder temperature. As demonstrated above, menthol did reduce licking behavior at 7°C. This demonstrates that the combination of cold temperatures and menthol do reduce licking behavior. These data support our earlier conclusions that menthol is reducing licking behavior at 21°C when compared to 33°C as other cold temperatures also reduce this behavior. The Lick/Face ratio also decreased significantly and these results are similar to our previous results involving menthol on cold temperature on an operant orofacial pain assay [17].

Capsaicin also altered licking behavior, but did not alter the Lick/Face ratio as we had expected. This lack of an effect of capsaicin on the Lick/Face is different from our previous reports [18], [19] so there is the possibility that this ramping protocol is having an effect on the behavior. The 3 min time period at 45°C is different from our previous studies in which the entire testing session is at a single temperature. As a result, our rats may be somewhat satiated already and less willing to endure the heat stimulus in order to access the reward. Modifying the ramping time periods in the future may alleviate this issue. Nonetheless, it appears there is a floor effect at 45°C such that capsaicin application does not significantly reduce the Lick/Face ratio when compared to baseline measures. Although the total number of licks was lower with capsaicin application compared to menthol application, the relationship between the temperatures was similar. 21°C and 45°C were equally inhibiting while the most licking occurred at 33°C. We had hypothesized that capsaicin would increase responding at 21°C as the lower temperature would “cool” the “burning” effect. We also hypothesized that it would reduce the licks at 45°C much more, but this does not seem evident. This could be due capsaicin's ability to produce mechanical allodynia [34]–[37]. If mechanical allodynia was occurring then this would reduce behavior at all three temperatures. The fact that the mean Lick/Face ratio at all three temperatures was reduced from baseline measures supports this hypothesis.

We pursued the hypothesis that altering the ramping protocol could explain these results. We placed the 45°C time period immediately after the first 33°C portion and examined behavior after capsaicin application as compared to baseline. Similar to our previously published results, capsaicin did decrease the Lick/Face ratio and licking behavior at this hot temperature [18], [19]. This demonstrates that the ramping protocol can alter pain behavior but further experiments will need to be performed to determine what the cause of this change is. One possibility is that the 21°C portion was sensitizing the rats and making the 45°C more painful than after a 33°C period. Another reason could be due to satiation. The rats have had 10 min of access to the reward solution by the time the 45°C occurs and therefore may not have sufficient motivation to respond at this nociceptive temperature. More discussion of the need to keep motivation constant over the course of the session and our recommendation for shorter behavioral protocols can be found in Anderson et al, [21]. As illustrated in the representative figures above (Figures 1D, 2D, 3D, and 4D), the rats to return to high levels of consumption during the final 3 min period of 33°C after both of the nociceptive sessions have finished which argues against the satiation hypothesis. Additional experiments will have to be performed to determine which, if either, of these hypotheses is valid.

In the final experiment, menthol and capsaicin were co-applied and the operant task was performed again. The Lick/Face ratio was similar to the previous conditions. Neither menthol alone, capsaicin alone, nor menthol and capsaicin together altered the nociceptive properties of the different temperatures. The Lick/Face was always highest at 33°C and lowest at 21°C and 45°C. We had hypothesized that menthol may reduce the painful effects of capsaicin, but instead our results were similar to a study in humans where a menthol pretreatment was demonstrated to be unable to block capsaicin induced decreases in heat pain thresholds [13]. Interestingly, the combination of menthol and capsaicin restored the original relationship for consumption behavior as now the number of licks was equal between the 33°C and 21°C periods again. This could be due to menthol being able to block some of the effects of capsaicin at this temperature similar to the “cooling” effect of menthol on hot temperatures [13]. So while a capsaicin and menthol co-application is still nociceptive, the inhibition of consumption behavior during the 21°C temperature is lessened.

Another possible explanation for the results obtained for the co-application experiment is that a form of cross-desensitization is occurring between menthol and capsaicin. When capsaicin and menthol are given at the same time orally to human volunteers, a short-term desensitization to the irritating effects of capsaicin was observed. This was attributed to cross-desensitization and could be playing a role in our study although several differences like time frame of measurement, method of chemical administration, and species tested make comparisons difficult [38]. These results were different than those observed in a similar study when either oral capsaicin or menthol was applied first followed by a 15 min wait period then followed by either capsaicin or menthol application [39]. This brings up the question of whether or not we would obtain the same results if menthol and capsaicin were applied one after another before starting our ramping protocol. The co-application could produce an analgesic effect whereas capsaicin before menthol could produce a desensitization to menthol and menthol pre-treatment could cause a cross-sensitization to capsaicin as observed in human volunteers [39]. We expect that future studies using a variety of application methods would reveal similar results to those reported by humans as our operant assay allows the rodent to inform the experimenter of the level of nociception it feels based on its decision to endure the nociceptive stimuli in order to obtain reward. More discussion of this concept is provided in Anderson et al.[21]. This cross-sensitization could be the result of interactions between TRPV1 and TRPM8 or TRPA1 [40]–[42]. Although this study does provide evidence that menthol induces changes in cold nociception and/or aversion, it is unknown which receptor(s) this effect is induced through. There is a debate in the literature concerning TRPM8, TRPA1, and cold nociception so more experiments would have to be performed, possibly with more selective agonists/antagonists (ex. mustard oil for a more specific TRPA1 but not TRPM8 effect) or TRPM8/TRPA1 knockout mice to tease out the exact mechanisms responsible for menthols effects on this operant behavior [43]–[58].

Finally, this paper highlights the ability of the OPAD to distinguish subtle changes in nociceptive behavior in rodents. Operant testing of pain has been difficult in the field of pain research, but the integration of our approach may help to clear up some results in the field in several ways. One benefit of this approach is that the nociceptive stimuli are not experimenter evoked. The rodent has the ability to control the level of nociception it is exposed to. This reduces the possible confounds of experimenter bias and variability that are found with more traditional pain measures like the tail-flick and von Frey tests. The operant boxes are also uniform and provide white noise which reduces the differences in the ambient environment found between laboratories as well. Our approach also reduces issues that can be found when the experimenter needs to be blinded to the groups as the behavior is analyzed identically and the nociceptive stimuli are changed automatically to preprogrammed levels. This will be especially important for genetic studies involving pain where it is almost impossible to be blinded to genotypes especially when there is an obvious phenotype. For more discussion of this topic we suggest our recently published methods paper [21].

In conclusion, rats find temperatures of 45°C and 21°C nociceptive and/or aversive, but rats consume more reward solution at 21°C than 45°C suggesting that the cool temperature is less aversive than the hot temperature. When either menthol or capsaicin is added alone, consumption behavior is equivalent between these two temperatures, likely due to capsaicin reducing cold nociception and menthol reducing heat nociception. The combination of menthol and capsaicin at the same time somewhat negates these effects as rats once again find 21°C to be less aversive than 45°C as they consume equal amounts of reward at 21°C and 33°C. As TRPM8/TRPA1 and TRPV1 agonists can modulate each other's effects on nociceptive behaviors, they could be useful in the modulation of certain types of pain in humans.
